# Protein kinase C*δ* expression in breast cancer as measured by real-time PCR, western blotting and ELISA

**DOI:** 10.1038/sj.bjc.6604728

**Published:** 2008-10-28

**Authors:** E McKiernan, K O'Brien, N Grebenchtchikov, A Geurts-Moespot, A M Sieuwerts, J W M Martens, V Magdolen, D Evoy, E McDermott, J Crown, F C G J Sweep, M J Duffy

**Affiliations:** 1 Department of Pathology and Laboratory Medicine, St Vincent's University Hospital, Dublin, Ireland; 2 UCD School of Medicine and Medical Science, Conway Institute of Biomolecular and Biomedical Research, University College Dublin, Dublin, Ireland; 3 Department of Chemical Endocrinology, Radboud University Nijmegen Medical Centre, Nijmegen, The Netherlands; 4 Department of Medical Oncology, Erasmus MC, Rotterdam, The Netherlands; 5 Frauenklinik der Technischen Universitaet München, Klinikum rechts der Isar, Munich, Germany; 6 Department of Medical Oncology, St Vincent's University Hospital, Dublin, Ireland

**Keywords:** breast cancer, protein kinase C*δ*

## Abstract

The protein kinase C (PKC) family of genes encode serine/threonine kinases that regulate proliferation, apoptosis, cell survival and migration. Multiple isoforms of PKC have been described, one of which is PKC*δ*. Currently, it is unclear whether PKC*δ* is involved in promoting or inhibiting cancer formation/progression. The aim of this study was therefore to investigate the expression of PKC*δ* in human breast cancer and relate its levels to multiple parameters of tumour progression. Protein kinase C*δ* expression at the mRNA level was measured using real-time PCR (*n*=208) and at protein level by both immunoblotting (*n*=94) and ELISA (*n*=98). Following immunoblotting, two proteins were identified, migrating with molecular masses of 78 and 160 kDa. The 78 kDa protein is likely to be the mature form of PKC*δ* but the identity of the 160 kDa form is unknown. Levels of both these proteins correlated weakly but significantly with PKC*δ* concentrations determined by ELISA (for the 78 kDa form, *r*=0.444, *P*<0.005, *n*=91 and for the 160 kDa form, *r*=0.237, *P*=0.023, *n*=91) and with PKC*δ* mRNA levels (for the 78 kDa form, *r*=0.351, *P*=0.001, *n*=94 and for the 160 kDa form, *r*=0.216, *P*=0.037, *n*=94). Protein kinase C*δ* mRNA expression was significantly higher in oestrogen receptor (ER)-positive compared with ER-negative tumours (*P*=0.007, Mann–Whitney *U*-test). Increasing concentrations of PKC*δ* mRNA were associated with reduced overall patient survival (*P*=0.004). Our results are consistent with a role for PKC*δ* in breast cancer progression.

The protein kinase C (PKC) family of genes encode serine/threonine kinases that regulate proliferation, apoptosis, cell survival and migration (for review, see Jackson and Foster, 2004; Griner and Kazanietz, 2007). Currently, nine PKC genes are known to exist. The PKC isoforms are classified into three subfamilies based on their structural and enzymatic properties: classical PKCs (*α*, *β*1, *β*2 and *γ)* are activated by diacylglycerol (DAG) and calcium, novel PKCs (*δ, ε, η* and *θ)* are activated by DAG but are calcium-insensitive and atypical PKCs (*ζ* and *λ*/*ι*) respond to neither DAG nor calcium. Although, originally, PKCs were believed to be mostly promitotic (Griner and Kazanietz, 2007), more recent findings suggest that the effects of at least some PKCs may be isoenzyme and cell-type dependent (Jackson and Foster, 2004; Griner and Kazanietz, 2007).

One of the PKCs shown to exhibit enigmatic properties is PKC*δ* (Jackson and Foster, 2004). Depending on the model system investigated, PKC*δ* has been shown to act as either a positive or a negative regulator of tumour progression (Jackson and Foster, 2004). In most situations, however, PKC*δ* has been shown to be growth inhibitory or proapoptotic (Griner and Kazanietz, 2007). In these situations, PKC*δ* might be regarded as a tumour suppressor gene.

In one of the first studies on breast cancer, Kiley *et al* (1999) showed that PKC*δ* was involved in metastasis in a rat model. More recently, Martens *et al* (2005) reported that hypermethylation of the PKC*δ* gene in oestrogen receptor (ER)-positive breast cancer patients was associated with a favourable response to hormone therapy in patients with advanced breast cancer, whereas Assender *et al* (2007) found that elevated PKC*δ* protein was associated with endocrine sensitivity. In breast cell lines, on the other hand, tamoxifen-resistant subclones tend to have higher PKC*δ* protein expression than their parental tamoxifen-sensitive counterparts (Nabha *et al*, 2005; Assender *et al*, 2007).

The aim of this study was to carry out a more detailed study on PKC*δ* in human breast cancer, in particular to relate PKC*δ* expression to multiple parameters of tumour progression. Our results show that increased PKC*δ* expression is associated with poor outcome. This finding is consistent with a role for PKC*δ* in breast cancer progression.

## Materials and methods

### Selection of patients and patient treatment

The breast cancers investigated in this study were random samples submitted for routine histopathology at St Vincent's University Hospital, Dublin. The piece of tumour used for experimental purposes was dissected out by a histopathologist, snap-frozen in liquid nitrogen and stored at −80°C until use. Tissue samples were homogenised using a Mikro-Dismembrator (Braun Biotech T1 International, Melsungen, Germany). Table 1 summarises the characteristics of the breast carcinomas analysed. Samples were obtained with institutional board approval from St Vincent's University Hospital.

For relating PKC*δ* to patient outcome, both disease-free and overall survival were used as end points. Disease-free interval was defined as the time from date of initial diagnosis to the date of first recurrence/metastasis. Overall survival was defined as the time from initial diagnosis to death from any cause or date of last follow-up. The median patient follow-up period was 34.8 months (0–137). Thirty-nine patients (18.8%) received adjuvant polychemotherapy, 50 (24%) received monohormone therapy and 107 (51.4) received combined chemotherapy and hormonal therapy. Two patients were not given any adjuvant systemic therapy, whereas for 10 patients, the adjuvant treatment was unknown.

### RNA isolation and real-time PCR

RNA was extracted from tumour samples using a commercially available column-based method (RNeasy® Mini kit; Qiagen, Hilden, Germany) and cDNA synthesised using Superscript II (Invitrogen, Carlsbad, CA, USA). The quality and quantity of the RNA was assessed using gel electrophoresis and spectrophotometery. RNA was DNase I treated (DNase I; NEB, Ipswich, MA, USA) before reverse transcription and cDNA was subsequently treated with RNAse H (NEB).

The expression of three housekeeping genes, that is, porphobilinogen deaminase (PBGD), hypoxanthine-guanine phosphoribosyltransferase (HPRT) and *β*-actin as well as PKC*δ* was measured using SYBR green-based real-time PCR (Roche LightCycler 2.0; Roche Diagnostics GmbH, Mannheim, Germany). The primer sequences for PKC*δ* were sense, TTCGGGAAGGTGCTGCTTG and antisense, TGCCCTTGCTGTGTAGAAAC, whereas those for *β*-actin were sense, GCACAGAGCCTCGCCTTTG and antisense CGCCCACATAGGAATCCTTC. The primer sequences for PBGD and HPRT were as previously described by Sieuwerts *et al* (2005). Each reaction mixture contained 20 ng of cDNA and a concentration of 0.33 *μ*M of each primer in a final volume of 20 *μ*l with Quantitect® SYBR green reaction master mix (Qiagen and Roche Diagnostics GmbH). The optimised amplification protocol consisted of an initial denaturation step of 95°C for 15 min, followed by 40 amplification cycles at 95°C for 15 s, annealing at 60°C for 30 s and elongation at 72°C for 25 s. A constant temperature ramp of 20°C s^−1^ was used throughout each of the steps. Measurements of fluorescence were taken at the end of each cycle. The PCR products were melted by increasing the temperature from 60 to 95°C (0.1°C s^−1^). Finally, the samples and carousel were cooled to 40°C. Positive and negative controls were included in each run. The negative control lacked template, whereas the positive control was pooled RNA extracted from HeLa cells. Levels of the PKC*δ* gene, expressed relative to the housekeeping set (HPRT, *PBGD* and *β*-actin), were quantified as follows: mRNA target=*E*^(mean *C*_t_ housekeeping−mean *C*_t_ target)^ (Sieuwerts *et al*, 2005).

### Immunisation

For PKC*δ*, two different 16-mer peptides (peptide PKCD2 (296–311) and peptide PKCD3 (314–329)), both located in the flexible linker region between the N-terminal regulatory and the C-terminal protein kinase domain, were synthesised (Pineda, Berlin, Germany), conjugated to KLH, and used for immunisations in chicken and rabbit. Immunisations were performed according to a time schedule with boosting intervals of 2 weeks. Chickens were immunised intramuscularly (pectoral muscle) with 120 *μ*g of conjugated peptide PKCD2. Eggs from the immunised chickens were collected daily and stored at 4°C.

Antibodies were isolated from egg yolk using a standard procedure of step precipitation of proteins by applying increasing concentrations of polyethyleneglycol (PEG precipitation) as described earlier (Grebenchtchikov *et al*, 2002). This procedure yielded IgY fractions (avian analogue of IgG) with a protein purity of approximately 95% (SDS–PAGE). The first injection (Freund's complete adjuvant) (PKCD3) in rabbit was in the popliteal gland, whereas booster injections (Freund's incomplete adjuvant) were administered subcutaneously with 2 weeks interval. In total, 10 booster injections were administered. Blood from rabbits was collected just before the inoculations in citrate tubes. After centrifugation, the citrate plasmas were stored at −20°C.

For immunoblotting, unpurified rabbit anti-PKCD3 polyclonal antibodies (V35) were used, whereas for ELISA chicken, anti-PKCD2 and rabbit anti-PKCD3 antibodies were affinity purified (Grebenschikov *et al*, 1997). Polyclonal antibodies were eluted from the columns with Gly/HCl buffer (pH 2.40), followed by immediate neutralisation to pH 7.5. Purified antibodies were characterised using ‘one-side ELISA’ methodology in which peptide fragments were coupled to the microtitre well. Specificity of the antibodies was verified by western blot analysis, which demonstrated a strong reaction with recombinant full-length PKC*δ* and truncated PKC*δ*, encompassing the N-terminal regulatory domain, but no reaction with the closely related recombinant PKC*γ*.

### Protein isolation and immunoblot analysis

Protein was extracted from tissue samples using 50 mmol l^−1^ Tris-HCl (pH 7.4) containing protease inhibitor cocktail (Roche Diagnostics GmbH) and Triton X-100 (1%) under agitation at 4°C for 1 h. A bicinchoninic acid assay (Pierce, Rockford, IL, USA) was used to determine total protein concentration. Equal amounts of protein were separated on SDS–PAGE and transferred to nitrocellulose membranes (Sigma, St Louis, MO, USA). Membranes were blocked in 5% low-fat dry milk (Marvel instant-dried skimmed milk) in TBS-T for 1 h at room temperature and then stained overnight with mouse anti-PKC*δ* monoclonal antibody (0.25 *μ*g ml^−1^; BD Biosciences Pharmingen, San Diego, CA, USA (catalogue no. 610 397)). Following three washes for 10 min in TBS-T, the membrane was incubated with 1 : 1000 horseradish peroxidase-conjugated anti-mouse secondary antibody (Sigma) for 1 h at room temperature before incubation with 6 ml of chemiluminescence reagent (Luminol; Santa Cruz Biotechnology, Santa Q7 Cruz, CA, USA) for 1 min. Membranes were exposed to X-ray film (Fujifilm) in the dark for 15 min. Positivity of expression was determined based on the presence of a visible band. The intensity of the protein bands observed was semi-quantified using the UVIBandMap programme (Windows application VIO.02), with normalisation of PKC*δ* protein against *β*-actin. Specificity of the antibody reaction was confirmed by (a) detection of the human recombinant PKC*δ* protein (Abcam, Cambridge, UK) at 78 kDa, which was used as a positive control, (b) omission of the primary antibody and (c) detection of a similar pattern of expression using a second primary antibody against PKC*δ* (see above for preparation of this antibody).

### ELISA

Protein kinase C*δ* concentrations were measured by ELISA with the same experimental setup as described earlier (Grebenschikov *et al*, 1997). The assay format incorporated four different antibodies: (a) duck anti-chicken IgY antibody, (b) chicken anti-PKCD2 antibody, (c) rabbit anti-PKCD3 and (d) goat anti-rabbit antibody labelled with HRP. The procedure started by treating the microtitre plates with coating antibody (duck anti-chicken IgY, overnight at 4°C), followed by blocking with BSA (2 h at 37°C) and incubation with capture antibody (chicken anti-PKCD2) for 2 h at 37°C. Incubation with the cancer extracts, reference sample and the standards was overnight at 4°C. For calibration, recombinant PKC*δ* preparation provided by Cell Sciences (Canton, MA, USA) was used. Incubation with trapping antibody (rabbit anti-PKCD3) as well as the subsequent incubation with detection antibody (goat anti-rabbit labelled with HRP) was performed for 2 h at ambient temperature. The incubation with OPD substrate solution was performed in darkness for 30 min at ambient temperature. The reaction was stopped by the addition of H_2_S0_4_ and the optical density was measured at 492 nm within 30 min. The analytical sensitivity, defined as the amount of PKC*δ* giving a signal in the ELISA greater than two standard deviations above blank values, was 0.5 ng ml^−1^. For estimation of the accuracy, a cytosolic reference preparation (labelled preparation 311 000) was used. The mean PKC*δ* concentration in preparation 311 000 was 28.4 ng ml^−1^, whereas the within-run coefficient of variation (CV) and the between-run CV were 3.1% (*n*=8) and 4.1% (*n*=7), respectively.

### Statistics

The Spearman rank correlation was used to compare continuous variables. The Mann–Whitney *U*-test, Kruskal–Wallis test (for continuous/nominal variables) and *χ*^2^ test (for nominal data) were used to determine relationships between the various variables. Computations were carried out using SPSS v.11 (SPSS Inc. Headquarters, Chicago, IL, USA). A two-sided *P*<0.05 was considered statistically significant. Protein kinase C*δ* mRNA was related to patient outcome using the Cox regression model. For this analysis, PKC*δ* mRNA level was treated as a continuous variable.

## Results

### Protein kinase C*δ* expression in breast carcinomas

**Figure 1:**
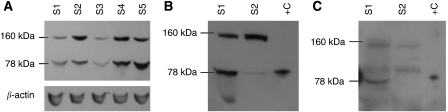
(**A**) Representative immunoblot of PKC*δ* expression in five primary breast carcinoma samples (S1–S5). Loading control, *β*-actin. (**B** and **C**) Confirmation of antibody specificity. Protein kinase C*δ* expression was examined in two primary breast carcinoma samples S1 and S2 using the mouse anti-PKC*δ* monoclonal antibody (BD Biosciences) (**B**) and the rabbit anti-PKCD3 polyclonal antibody (V35) (**C**). The same carcinoma samples were used in (**B**) and (**C**). Positive control, human recombinant PKC*δ* protein (+C).

Protein kinase C*δ* mRNA was measured by real-time PCR in 208 breast carcinoma samples (see Table 1 for clinical characteristics of the cohort). The median value relative to the housekeeping gene set was 1.97-fold with a range from undetectable to 17.3-fold. Ninety-four representative samples of the 208 analysed by RT–PCR were subjected to immunoblotting for PKC*δ* (see also Table 1 for clinical characteristics of this subgroup). Figure 1A illustrates a representative immunoblot of PKC*δ* protein expression, following electrophoresis under reducing conditions. Using a mouse anti-PKCδ monoclonal antibody (BD Biosciences Pharmingen), two bands were identified, migrating with molecular masses of ∼160 and 78 kDa. On the basis of its known molecular mass, the 78 kDa band was regarded as the parent form of PKC*δ* (Emoto *et al*, 1995). To our knowledge, a 160 kDa form of PKC*δ* has not been previously described. Thus, the identity of this band is unknown. The 78 kDa protein was found in 72 out of 94 (76.6%) of the samples. Concentrations ranged from undetectable to 1.97 arbitrary units, the median value being 0.072 arbitrary units. On the other hand, the 160 kDa protein was detected in 86 out of 94 (91.5%) of the samples with concentrations ranging from 0 to 5.15 arbitrary units and a median value of 0.205 arbitrary units.

As a 160 kDa form of PKC*δ* had not previously been described, we decided to check its reactivity with a second antibody against PKC*δ*. Using a rabbit polyclonal antibody directed against peptide PKCD3, encompassing amino acids 314–329 of PKC*δ* (for details, see Materials and Methods), the 160 kDa band was also detected (Figure 1B and C), suggesting that this band may be either a new form of PKC*δ*, PKC*δ* complexed to an unknown protein or a protein closely related to PKC*δ*. To check the possibility that the 160 kDa band might be a dimer of the parental form of PKC*δ*, we increased the concentration of the reducing agent, that is *β*-mercaptoethanol, in the loading buffer from 5 to 20%. The pattern of expression for both the 78 kDa parent form and the 160 kDa forms was not altered, suggesting that the 160 kDa protein is unlikely to be a dimer.

**Figure 2:**
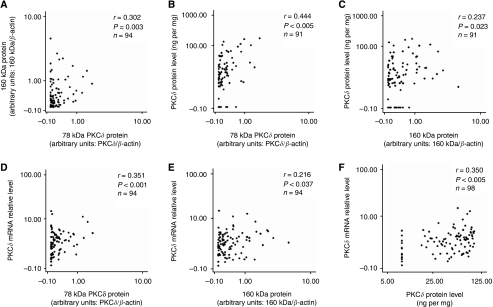
(**A**) Bivariate scattergram illustrating the relationship between the 78 kDa parent form of PKC*δ* and the 160 kDa form. (**B** and **C**) Relationship between PKC*δ* protein as measured by both ELISA and immunoblot. Bivariate scattergrams illustrating the positive correlations between PKC*δ* protein levels as measured by ELISA and (**B**) the 78 kDa PKC*δ* form, (**C**) the 160 kDa form. (**D** and **E**) Relationship between PKC*δ* mRNA and PKC*δ* protein as measured by real-time PCR and immunoblotting. Bivariate scattergrams illustrating the positive correlations between PKC*δ* mRNA and (**D**) the 78 kDa PKC*δ*; (**E**) the 160 kDa form. (**F**) Bivariate scattergram illustrating the positive correlation between PKC*δ* mRNA and total PKC*δ* protein as measured by ELISA. Scale on both axes is logarithmic. Analysis performed using the Spearman rank statistical test.

Using the Spearman rank correlation test (Figure 2A), a weak but significant correlation was found between the 78 kDa parent form of PKC*δ* and the 160 kDa protein in the carcinomas (*r*=0.302, *r*^*2*^=0.081, *P*=0.003, *n*=94). Of the 94 samples investigated, 70 (74.5%) were positive for both proteins, whereas six (6.4%) were negative for both forms. Two samples (2.1%) expressed the 78 kDa form in the absence of the 160 kDa form. Sixteen of the samples (17%) expressed the 160 kDa form in the absence of the 78 kDa band.

Extracts from 98 samples of the 208 analysed by RT–PCR were assayed for PKC*δ* by ELISA. Using ELISA, the total PKC*δ* protein concentration ranged from undetectable to 176.0 ng per mg protein, with the median value being 10.5 ng per mg protein. A significant but weak correlation was found between the levels of PKC*δ* as determined by ELISA and both the 78 kDa form of PKC*δ* and the 160 kDa protein determined by immunoblotting (for the 78 kDa form, *r*=0.444, *r*^*2*^=0.187, *P*<0.005, *n*=91 and for the 160 kDa form, *r*=0.237, *r*^*2*^=0.046, *P*=0.023, *n*=91; Figure 2B and C).

As shown in Figure 2D and E, a significant but weak correlation was found between PKC*δ* mRNA expression and both the 78 kDa PKC*δ* protein and the 160 kDa band (for the 78 kDa form, *r*=0.351, *r*^*2*^=0.114, *P*=0.001, *n*=94 and for the 160 kDa band, *r*=0.216, *r*^*2*^=0.036, *P*=0.037, *n*=94). Protein kinase C*δ* mRNA expression also correlated with PKC*δ* protein, as determined by ELISA (*r*=0.350, *r*^*2*^=0.113, *P*<0.005, *n*=98; Figure 2F).

### Relationship between PKC*δ* expression and both tumour characteristics and patient outcome

**Figure 3:**
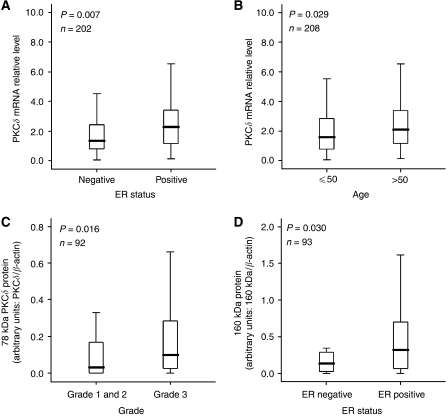
Box plot representing the relationship between (**A**) PKC*δ* mRNA levels and ER status in primary breast carcinomas; (**B**) PKC*δ* mRNA levels and patient age at the time of diagnosis; (**C**) the 78 kDa PKC*δ* protein form as measured by immunoblot and tumour grade and (**D**) the 160 kDa form as measured by immunoblot and ER status in primary breast carcinomas. Boxes, 25th and 75th percentiles with the median indicated; bars, 10th and 90th percentiles. Analysis was performed using the Mann–Whitney *U*-test.

Protein kinase C*δ* mRNA and protein levels were related to established prognostic factors for breast cancer, that is tumour size, presence or absence of axillary node metastasis, tumour grade, histology type, ER status and patient age at time of diagnosis (Table 2). No significant correlation was found between PKC*δ* expression and tumour size, and the presence or absence of axillary node metastasis or histology type. Levels of PKC*δ* mRNA, however, were significantly higher in ER-positive tumours compared with ER-negative tumours (Mann–Whitney *U*-test: *P*=0.007, *n*=202; Figure 3A) and in patients older than 50 years at diagnosis than in those younger than 50 years (Mann–Whitney *U*-test: *P*=0.029, *n*=208; Figure 3B). Levels of the 78 kDa form of PKC*δ*, but not the 160 kDa protein, were significantly elevated in high-grade tumours compared with low-grade tumours (Mann–Whitney *U*-test: *P*=0.016, *n*=92; Figure 3C). Levels of the 160 kDa protein, but not the 78 kDa protein, were significantly elevated in ER-positive tumours compared with ER-negative tumours (Mann–Whitney *U*-test: *P*=0.030, *n*=93; Figure 3D).

Protein kinase C*δ* mRNA expression was related to both patient disease-free interval and overall survival. Although increasing levels of PKC*δ*, treated as a continuous variable, tended to correlate with shorter disease-free interval, this relationship failed to reach statistical significance (*P*=0.074) (Table 3). However, the increasing expression of PKC*δ* was associated with a significantly shorter overall survival (*P*=0.004) (Table 3). Other factors associated with shortened survival included tumour size (treated as a continuous variable) (*P*=0.015), tumour grade (grade 3 *vs* grades 1 and 2 combined) (*P*=0.009) and the number of lymph nodes with metastasis (*P*=0.002). Using multivariate analysis, PKC*δ* mRNA predicted overall survival, independent of the traditional prognostic factors (Table 3). Protein kinase C*δ* protein levels were not related to patient outcome as the number of samples analysed at the protein level was too low and we did not have enough events (i.e., recurrences or deaths) to provide reliable associations with outcome.

## Discussion

This is one of the most comprehensive studies published to date on PKC*δ* in a large cohort of human breast cancers. Our results show that this gene is variably expressed at both the mRNA and protein levels in breast cancer and that PKC*δ* protein levels in breast cancer specimens, as measured by immunoblotting and ELISA, correlate weakly but significantly with each other and with PKC*δ* mRNA levels. At both mRNA and protein levels, the expression of PKC*δ* was independent of tumour size, lymph node status and histology type. Levels of PKC*δ* mRNA were, however, significantly higher in ER-positive compared with ER-negative patients. This finding suggests a possible relationship between PKC*δ* expression and endocrine sensitivity in patients with breast cancer. In this context, it is of interest to note that Martens *et al* (2005) found that hypermethylation of the PKC*δ* gene in ER-positive patients was associated with a favourable response to tamoxifen therapy in advanced breast cancer. Although the effect of hypermethylation on the expression of PKC*δ* mRNA was not investigated in that study, gene hypermethylation is generally, but not universally, associated with a decreased expression (Branscombe and Jones, 2007). While this work was in progress, Assender *et al* (2007) reported that elevated PKC*δ* protein, as determined by immunohistochemistry, was associated with endocrine sensitivity in patients with advanced breast cancer.

As only a minority of the patients in this study were treated with hormone monotherapy, it was not possible to investigate the relationship between PKC*δ* expression and outcome following such a treatment. Rather, we found that elevated expression of PKC*δ* was associated with poor overall survival. As most of our patients receive systemic adjuvant therapy, it was not possible to conclude whether PKC*δ* was prognostic, predictive or both. In any event, our finding of a significant relationship between increased PKC*δ* expression and shortened overall survival is consistent with previous studies from a model system, implicating this kinase in breast cancer metastasis (Kiley *et al*, 1999).

The association between high expression of PKC*δ* and shortened overall survival may appear to be inconsistent with our finding of a positive relationship between the kinase and ER (see above). This is because ER-positive breast cancer patients are generally regarded to have a better prognosis than ER-negative patients (Duffy, 2006). The favourable prognostic impact of ER, however, depends on the adjuvant therapy administered. Thus, in a study on 2562 patients, ER was of prognostic value only in the subgroup treated with adjuvant tamoxifen therapy (Khoshnoud *et al*, 2008). In our study, only 24% of the patients received hormone monotherapy, whereas most received combined hormone and chemotherapy. Indeed, ER was not a significant predictor of patient outcome in our group of patients.

Our finding of a significant relationship between PKC*δ* and overall survival, but not between the kinase and disease-free interval, may relate to the fact that almost all our patients received some form of systemic adjuvant therapy. A potential adverse prognostic impact of PKC*δ* could thus be neutralised or partially neutralised by this adjuvant treatment. It should also be added that while the association between PKC*δ* and disease-free interval did not reach statistical significance, there was a trend towards significance, that is the *P*-values were 0.074 and 0.052 using univariate and multivariate analyses, respectively.

Following immunoblotting with two different antibodies against PKC*δ*, we identified two main proteins, migrating with molecular masses of 78 and 160 kDa. On the basis of its known molecular mass, the 78 kDa band was taken to represent the mature form of PKC*δ*. To our knowledge, a 160 kDa form of PKC*δ* has not previously been described. Further work is therefore required to establish whether it is a new form of PKC*δ*, a protein related to PKC*δ*, a complex of PKC*δ* with an unknown protein or indeed a non-specific band.

The 78 kDa form of PKC*δ* was expressed at higher levels in high-grade (that is, grade 3) compared with low-grade (that is, grade 1 and 2) tumours. Grade is a well-established prognostic factor for breast cancer with increasing grade generally being associated with poorer outcome (Soerjomataram *et al*, 2008). The finding of a positive relationship between levels of the 78 kDa form of PKC*δ* and grade is consistent with our finding that elevated expression of PKC*δ* mRNA was associated with poor outcome.

As mentioned in the introduction, there is considerable debate at present as to whether PKC*δ* is involved in promoting or inhibiting cancer progression. Our finding here of an association between elevated PKC*δ* mRNA expression and poor outcome suggests a role for PKC*δ* in tumour progression, at least in human breast cancer. Further studies, ideally using prospective randomised trials, are required to establish whether PKC*δ* has prognostic or predictive roles in breast cancer.

## Figures and Tables

**Table 1:** Pathological features and ER status of the breast carcinomas used

**Tumour characteristics**	**PCR *n* (%)**	**Western blot *n* (%)**	**ELISA *n* (%)**
*Size (cm)*			
⩽2	53 (25.5)	20 (21.3)	20 (20.4)
>2–5	127 (61.1)	62 (66.0)	62 (63.2)
>5	15 (7.2)	8 (8.5)	8 (8.2)
Unknown	13 (6.2)	4 (4.2)	8 (8.2)
			
*Grade*			
1 and 2	98 (47.1)	38 (40.4)	38 (38.8)
3	100 (48.1)	54 (57.5)	57 (58.2)
Unknown	10 (4.8)	2 (2.1)	3 (3.0)
			
*Nodal status*			
Negative	87 (41.8)	43 (45.7)	41 (41.8)
Positive	109 (52.4)	48 (51.1)	53 (54.1)
Unknown	12 (5.8)	3 (3.2)	4 (4.1)
			
*ER*			
Negative	51 (24.5)	24 (25.5)	24 (24.5)
Positive	151 (72.6)	69 (73.4)	73 (74.5)
Unknown	6 (2.9)	1 (1.1)	1 (1.0)
			
*Age*			
⩽50	65 (31.2)	24 (25.5)	28 (28.6)
>50	143 (68.8)	70 (74.5)	70 (71.4)
			
*Histological type*			
Ductal	173 (83.2)	83 (88.3)	86 (87.8)
Lobular	19 (9.1)	6 (6.4)	7 (7.1)
Ductal and lobular	11 (5.3)	3 (3.2)	3 (3.1)
Unknown	5 (2.4)	2 (2.1)	2 (2.0)

ER=oestrogen receptor.

**Table 2:** Relationship between PKC*δ* expression and characteristics of the breast carcinomas

	**mRNA (PCR)**			**Protein (western blot)**					**Protein (ELISA)**		
				**78 kDa isoform**			**160 kDa isoform**				
**Tumour characteristics**	***n***	**Median**	***P*-value**	***n***	**Median**	***P*-value**	**Median**	***P*-value**	***n***	**Median**	***P*-value**
*Size (cm)*											
⩽2	53	1.75	0.406^*^	20	0.0288	0.139^*^	0.2155	0.989^*^	20	10.56	0.635^*^
>2–5	127	2.04		62	0.0896		0.2051		62	12.49	
>5	15	1.43		8	0.0367		0.2576		8	8.48	
											
*Grade*											
1 and 2	98	2.13	0.613	38	0.0315	0.016	0.2024	0.883	38	7.88	0.243
3	100	1.8		54	0.1003		0.1968		57	12.80	
											
*Nodal status*											
Negative	87	1.98	0.874	43	0.0991	0.401	0.1555	0.290	41	11.68	0.580
Positive	109	1.89		48	0.0663		0.2981		53	9.57	
											
*ER*											
Negative	51	1.34	0.007	24	0.0836	0.349	0.1369	0.030	24	14.30	0.977
Positive	151	2.28		69	0.0626		0.3236		73	9.57	
											
*Age*											
⩽50	65	1.97	0.029	24	0.0744	0.767	0.2335	0.900	28	7.82	0.392
>50	143	2.54		70	0.0685		0.2		70	11.45	
											
*Histological type*											
Ductal	173	1.88	0.250^*^	83	0.0742	0.889^*^	0.2104	0.891^*^	86	9.71	0.336^*^
Lobular	19	2.15		6	0.1447		0.0917		7	20.87	
Ductal and lobular	11	2.89		3	0.062		0.1792		3	26.88	

ER=oestrogen receptor; PKC=protein kinase C.

*P*-values were determined by either the Mann–Whitney *U*-test or Kruskall–Wallis test^*^.

**Table 3:** Relationship between PKC*δ* mRNA and both overall survival and disease-free interval in breast cancer

	**Univariate analysis**	**Multivariate analysis**		
**Factors**	***P*-value**	***P*-value**	**RR**	**95% CI**
*Overall survival*				
Tumour size (continuous)	0.015	0.172	1.206	0.922–1.578
Nodal category^a^	0.002	0.037	1.996	1.044–3.816
Tumour grade (3 *vs* 1 and 2)	0.009	0.079	2.796	0.887–8.821
ER status	0.615	NS^b^		
PKC*δ* (continuous)	0.004	0.038	1.157	1.008–1.328
				
*Disease-free interval*				
Tumour size (continuous)	0.161	NS^b^		
Nodal category^a^	0.001	0.021	1.871	1.098–3.189
Tumour grade (3 *vs* 1 and 2)	0.010	0.074	2.370	0.919–6.110
ER status	0.977	NS^b^		
PKC*δ* (continuous)	0.074	0.052	1.125	0.999–1.268

CI=confidence interval; PKC=protein kinase C; RR=relative risk.

a Nodal status divided into categories: 0, 1–3 and >3 involved nodes.

b NS, not significant (*P*>0.05).
